# Pathogenic potential of bacteria isolated from commercial biostimulants

**DOI:** 10.1007/s00203-022-02769-1

**Published:** 2022-02-04

**Authors:** Daniela Bulgari, Silvia Filisetti, Matteo Montagna, Emanuela Gobbi, Franco Faoro

**Affiliations:** 1 Agri-Food and Environmental Microbiology Platform (PiMiAA), Department of Molecular and Translational Medicine, University of Brescia, viale Europa, 11, 25123 Brescia, Italy; 2 Department of Agricultural and Environmental Sciences - Production, Landscape, Agroenergy, University of Milan, via Celoria 2, 20133 Milan, Italy; 3 BAT Center - Interuniversity Center for Studies On Bioinspired Agro-Environmental Technology, University of Napoli “Federico II”, Portici, Italy; 4 Department of Agricultural Sciences, University of Naples “Federico II”, Via Università 100, Portici, 80055 Naples, Italy

**Keywords:** Sustainable agriculture, *Bacillus*, *Franconibacter*, Swimming, Swarming

## Abstract

Microbial-based products are a promising alternative to agrochemicals in sustainable agriculture. However, little is known about their impact on human health even if some of them, i.e., *Bacillus* and *Paenibacillus* species, have been increasingly implicated in different human diseases. In this study, 18 bacteria were isolated from 2 commercial biostimulants, and they were genotypically and phenotypically characterized to highlight specific virulence properties. Some isolated bacteria were identified as belonging to the genus *Bacillus* by BLAST and RDP analyses, a genus in-depth studied for plant growth-promoting ability. Moreover, 16S rRNA phylogenetic analysis showed that seven isolates grouped with *Bacillus* species while two and four clustered, respectively, with *Neobacillus* and *Peribacillus.* Unusually, bacterial strains belonging to *Franconibacter* and *Stenotrophomonas* were isolated from biostimulants. Although *Bacillus* species are generally considered nonpathogenic, most of the species have shown to swim, swarm, and produced biofilms, that can be related to bacterial virulence. The evaluation of toxins encoding genes revealed that five isolates had the potential ability to produce the enterotoxin T. In conclusion, the pathogenic potential of microorganisms included in commercial products should be deeply verified, in our opinion. The approach proposed in this study could help in this crucial step.

**Supplementary Information:**

The online version contains supplementary material available at 10.1007/s00203-022-02769-1.

## Introduction

Microbial products represent a promising alternative to agrochemicals in a sustainable agriculture either as biopesticides or biofertilizers or biostimulants (Bulgari and Faoro 2018; Bulgari et al. 2019; Pellegrini et al. 2020). Recently, the European Parliament defined plant biostimulant as ‘a product stimulating plant nutrition processes independently of the product’s nutrient content with the sole aim of improving one or more of the following characteristics of the plant and its rhizosphere: nutrient use efficiency, tolerance to abiotic stress, quality traits and availability of confined nutrients in soil or rhizosphere’ (European Union. 2019). Biostimulants are composed mainly by protein hydrolysates and other N-containing compounds, seaweed extracts, chitosan, humic, and fulvic acids, plant growth-promoting bacteria, and fungi (Yakhin et al. 2017). Microorganisms can enhance plant growth directly by phosphate solubilization, atmospheric nitrogen fixation, iron chelation, and production of secondary metabolites or indirectly by protecting plant from pathogens deleterious effects (Hardoim et al. 2015). To date, few genera are formulating for plant growth promotion or pest and pathogens control. *Bacillus* is a species-rich genus of Gram-positive rod-shape bacteria inhabiting different environments such as soil, water, and plants. Several strains of the species *Bacillus amyloliquefaciens*, *B. subtilis*, *B. pasteurii*, *B. cereus*, *B. pumilus*, *B. mycoides*, and *B. sphaericus* elicit significant reduction in the incidence or severity of various diseases in different hosts (Ruiu 2018). These results have been reported against fungal and bacterial pathogens, systemic viruses and root-knot nematodes (Choudhary and Johri 2009). *Bacillus* strains are characterized by heat- and desiccation-resistant spores, which can be easily formulated as stable dry powder, granules or liquid. *Bacillus thuringiensis* is present in almost 90% of biopesticides market in USA and in EU (Arthurs and Dara 2019). In Italy, *Bacillus*-based biostimulants represent the 42% of the market products. Bacteria belonging to this genus have an impact on human activities, ranging from human health care to agriculture. The most in-depth studied species among this group are the medically relevant *B. anthracis,* the causal agent of anthrax and *B. cereus* known to cause food-borne intoxication (Liu 2011). Little is known about the other species of the genus that are generally considered as contaminant when found in clinical cultures and their potential pathogenicity has been scarcely investigated (Celandroni et al. 2016). However, some reports indicate that these organisms can cause nosocomial infections or severe illness both in immunocompromised and immunocompetent humans, even if rarely (Idelevich et al. 2013; Abrishami et al. 2015).

Thus, the potential human pathogenicity of the *Bacillus* species, despite these bacteria are commonly considered soil-related organisms, should not be neglected, as they are increasingly isolated from hospitalized patients (Celandroni et al. 2016). In bacteria, the pathogenic potential is related to the presence of several virulence factors. Notably, in *B. cereus*, these include the secretion of virulence proteins several hemolysins, phospholipases, trimeric toxins (hemolysin BL, HBL; non-hemolytic enterotoxin, NHE), cytotoxin K (CytK) and proteases (Jessberger et al. 2019). In addition, also motility modes, such as swimming and swarming, and biofilm formation could confer pathogenic abilities (Senesi and Ghelardi 2010; Mazzantini et al. 2016). To our knowledge, no comprehensive studies have been carried out to evaluate the potential pathogenicity of microorganisms used as plant growth promoter (PGPR). The massive use of microorganisms without or poor knowledge of their impact on human health could lead to long-lasting consequences. For example, PGPR and biocontrol agent (BCA) can interact with human pathogens harbored by plants, possibly leading to horizontal gene transfer (i.e., resistance to antibiotics) (Van Overbeek et al. 2014; Holden et al. 2015; Bulgari et al. 2019). Moreover, PGPR could evolve new mechanisms for crossing the kingdom border and thus become true or opportunistic pathogens particularly dangerous in immunocompromised individual.

In this study, the bacteria present in commercial biostimulant products were characterized to evaluate their virulence potential by assessing motility and ability to form biofilms, as well as the presence of toxin-encoding genes.

## Materials and methods

### Bacteria isolation

Bacteria strains were isolated from three commercial biostimulants categorized, respectively, as fertilizer with plant action (FP) and fertilizer with soil action (FS) both in the subcategory inoculum with mycorrhizal fungus by the Italian Ministerial Decree 75/2010 (Table 1) by cultivation-dependent methods with and without microbe enrichment. Plant biostimulant was dissolved into Ringer Solution in 1:10 ratio. Then, 100 µL for each serial dilution were spread on Luria–Bertani (LB, Merck, Darmstadt, Germany), on Tryptic Soy Agar (TSA, Merck, Darmstadt, Germany) and on Potato Dextrose Agar (PDA, Merck, Darmstadt, Germany) plates and incubated at 30 °C (LB, TSA) or 24 °C (PDA) for 5 days.

**Table 1:** Commercial biostimulants’ details

Product	Composition	Concentration	Formulation	Dose
Fertilizer with action on plant	Mycorrhizae	0.10%	Microgranules	15 kg/ha
	*Trichoderma* sp.	1 × 10^5^ UFC/g		
	Rhizosphere bacteria	1 × 10^7^ UFC/g		
Fertilizer with action on soil	*Glomus intraradices* strain CMCCROC7	0.10%	Powder	1 kg/ha
	Rhizosphere bacteria	1 × 10 UFC/g		
Fertilizer with action on soil	Mycorrhizae	0.50%	Liquid	15 mL/20 L water
	*Trichoderma* sp.	1.3 × 10^7^ UFC/g		
	Rhizosphere bacteria	1.2 × 10^8^ UFC/g		

Two grams of each market biostimulants was dissolved into 200 mL Tryptic Soy Agar (TSB, Merck, Darmstadt, Germany) and shaken at 30 °C overnight for microbe enrichment. Partial volume (100 µL) of homogenates, serially diluted, was incubated on TSA at 30 °C for 5 days. Bacterial colonies were selected on the basis of phenotypic traits and isolated.

### Bacteria identification

Genomic DNA was extracted from pure culture using “DNeasy Blood & Tissue Kit” (Qiagen, Hilden, Germany) following manufacturer’s instruction. The bacterial 16S rRNA gene was amplified by 27F/1492r (Lane 1991; Chelius and Triplett 2001) following PCR conditions previously described (Bulgari et al. 2009); PCR products were sequenced using Microsynth (Microsynth AG, Balgach—CH) Sanger Sequencing Service. The obtained electropherograms were manually edited and checked using Geneious Pro R10 (Biomatters Ltd., Auckland, New Zealand). Sequences were compared with those contained into NCBI (National Center for Biotechnology Information) database using BLAST Nucleotide (https://blast.ncbi.nlm.nih.gov/Blast.cgi) selecting the option ‘Limit to Sequences from type material’ and the taxonomy tentatively assigned with RDP classifier (http://rdp.cme.msu.edu/classifier/classifier.jsp). To investigate the phylogenetic relationships of the isolated bacteria, 17 orthologous sequences to those amplified in the present study (16S rRNA) were retrieved from GenBank (https://www.ncbi.nlm.nih.gov/genbank/) for the following taxa: *Bacillus*, *Franconibacter*, *Stenotrophomonas*, *Neobacillus*, *Peribacillus* and of *Thermotoga maritima* NR029163 (considered as outgroup). The 16S rRNA sequences retrieved from GenBank plus those obtained in the present study (only one sequence per haplotype was retained) were aligned using MAFFT (Katoh et al. 2005), with E-INS-i iterative refinement methods. The selection of nucleotide evolution model best fitting the sequence alignment and the phylogenetic inference adopting Bayesian and Maximum Likelihood approaches were performed as previously described (Montagna et al. 2016). Briefly, jModelTest 2 (Darriba et al. 2012), with the adoption of the Akaike Information Criteria (Akaike 1973), was used to estimate and evaluate the model of nucleotide evolution; GTR (Lanave et al. 1984) + gamma distribution ($\Gamma$) + proportion of invariant sites (I) was selected as best model of nucleotide evolution. Bayesian analyses were performed using MrBayes 3.2 (Ronquist et al. 2012) with the following parameters: GTR + I+$\Gamma$ model of nucleotide evolution, two runs with four chains of 30 $\times$ 10^^6^ MCMC generations, thinning every 1,000 generations, and a burn-in fraction of 0.20. The ML analyses were performed using PhyML 3.0 (Guindon and Gascuel 2003) implementing GTR + I+$\Gamma$ model of nucleotide evolution, the best of NNI and SPR tree searching operation and computation of the starting tree with BioNJ. Node support was estimated by an approximate likelihood ratio test approach (aLRT) (Anisimova and Gascuel 2006). All trees were rooted on *T. maritima* (NR029163).

### PCR screening of toxin-encoding genes

The detection of virulence genes inside bacterial genome was verified by PCR amplification. Primers sequences and PCR conditions used to amplify *sph*, *bceT*, *entFM/S*, *piplc*, *cytK*, *nheA*, *nheB*, and *nheC* encoding sphingomyelinase, enterotoxin T and enterotoxin FM/S, PI-PLC, CytK, and the three components of NHE (Ghelardi et al. 2002), respectively, are reported in Supplementary Table 1.

PCRs were assembled in a final volume of 25 µL with 1 µL of the template DNA (10 ng/µL), 0.4 mM of each primer, 0.2 mM of dNTPs, and 2 units/µL of *Taq* polymerase (Promega, Madison, USA) in the supplied buffer. The DNA (10 ng/µL) extracted by *Bacillus cereus* ATCC 11,778 was used such as positive control.

### Motility test

Swimming and swarming motility were evaluated as previously described by Celandroni et al 2016. Briefly, for swimming motility assay, a volume of 1 µL of the overnight culture (1 × 10^8^ cells/mL) was seeded onto the center of TrM plates (1% tryptone, 0.5% NaCl, 0.25% agar), then plates were incubated at 30 °C and 37 °C in humidified chamber and the diameters of halos due to bacterial migration measured 24 h after the inoculation. For swarming motility assay 1 × 10^4^ cells/mL were spotted onto TrA (tryptone 1%, NaCl 0.5%, agar 0.7%) plates, incubating in humidified chamber at 30 °C and 37 °C and evaluated over time (24 h, 48 h and 96 h). Furthermore, the rims of growing colonies and of cells were analyzed by light microscope 24 h and 48 h after incubation, respectively.

### Light and electron microscopy

The morphology of the bacterial isolates was characterized by light interference contrast (DIC) microscopy using an Olympus BX50 light microscope equipped with a Retiga 2000R (Qimaging, USA) digital camera. Some isolates were also observed by a transmission electron microscope Jeol 100-SX (Jeol, Japan) by negative staining with 2% uranyl acetate.

### Biofilm formation

The ability of the bacterial strain to form biofilm was tested as previously described by Celandroni et al. (2016). LB overnight cultures of each bacterial strains were adjusted to an optical density of 0.01 at 610 nm (OD_610_) and incubated in 96-well plates at 37 °C for 8 h and 50 rpm shaking. The isolates were tested in duplicate. The total growth (OD_610_) in each well was measured; planktonic bacteria were removed, and the wells washed with distilled water and air-dried. Biofilms were stained with 200 µL of 0.3% crystal violet for 10 min, washed with distilled water, and air-dried. The crystal violet was solubilized with 200 µL of 70% ethanol and the OD_610_ was measured.

## Results and discussion

### Bacteria isolation and identification

Bacteria and fungi present in three commercial biostimulants were isolated by cultivation-dependent methods (Table 1). No microorganisms were isolated from the fertilizer with action on soil (FS) formulated in liquid. In detail, no *Trichoderma* species were cultivated even if they were reported on the label confirming that some biostimulants available in the market are of poor quality due to the absence of microorganisms or to the choice of an inappropriate carrier (Pathania et al. 2020). Moreover, the vitality of microorganisms could be affected by incorrect storage, handling and formulation (Singh and Nautiyal 2012; Pathania et al. 2020).

**Fig. 1:**
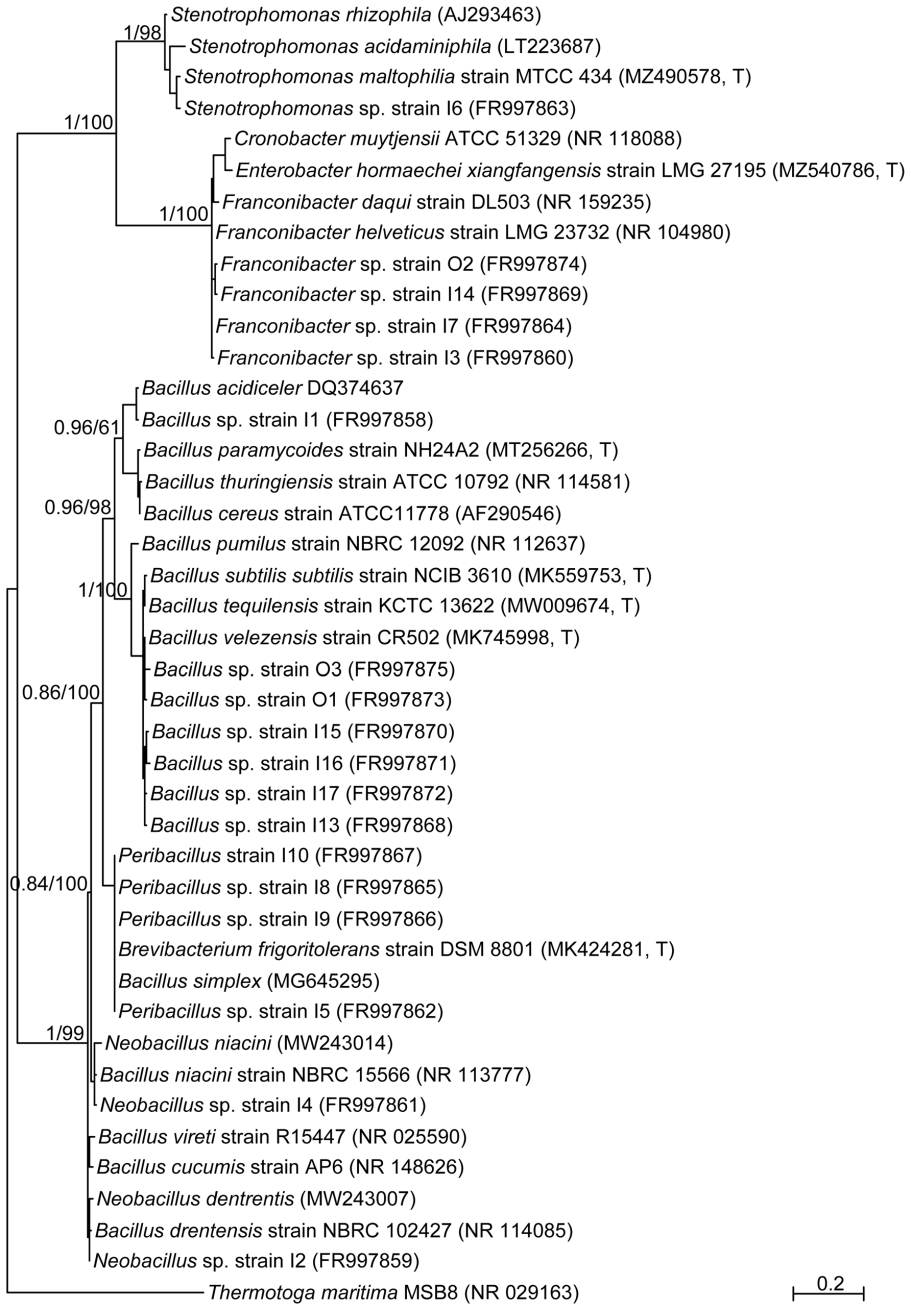
Bayesian phylogram inferred on 16S rRNA gene sequence obtained by the isolated bacteria plus those retrieved from public repositories (see “Materials and methods”). The values reported on the nodes of the main lineages are the Bayesian posterior probabilities and the aLRT values maximum-likelihood bootstrap percentages (values below 0.7 are not reported). The scale bar indicates the distance in substitutions per site

A total of 18 colonies with different morphology were isolated using LB and TSA. Fifteen isolates derived from Fertilizer with action on plant (FP) and 3 from Fertilizer with action on soil (FS) using enrichment method (Table 1). Five colonies, identified by BLAST and RDP analyses on partial 16S rRNA gene sequences, resulted as belonging to the class of Gammaproteobacteria and assigned to the genus *Franconibacter* (identity ≥ 97.85% with *Franconibacter* species) and *Stenotrophomonas* (identity ≥ 99.88% with cogenerics); 13 colonies belong to the phylum of Firmicutes and showed high identity values with members of the genus *Bacillus* (identity ≥ 99.28%), *Neobacillus* and *Peribacillus* (identity ≥ 97.68% and identity ≥ 99.31%, respectively) (Table 2)*.* As expected, *Bacillus* were the most abundant microorganisms resulting present in the commercial biostimulants, *Bacillus* is a heterogeneous group of bacteria with a different impact on human life ranking from severe illness, food poisoning, industrial and agriculture applications. *Bacillus* species are in deep studied biostimulants or biocontrol agents. Accordingly, five and two different *Bacillus* species were isolated from FP and FS biostimulants, respectively. In detail, *Bacillus subtilis* group is primarily sold as biocontrol agent to control plant pathogens (Dunlap 2019). Surprisingly, the genus *Stenotrophomonas* was isolated only from FP. Even if *Stenotrophomonas* species are isolated from rhizosphere and show antifungal activity (Jiang et al. 2021; Schmidt et al. 2021), *Stenotrophomonas* sp. strain I6 showed high similarity (identity ≥ 99.88%) with *Stenotrophomonas maltophilia.* This species is increasingly reported as an opportunistic and nosocomial pathogens (Alsuhaibani et al. 2021) and it shows resistance to different antibiotics such as carbapenems and fluoroquinolone (Harmon et al. 2019; Azimi et al. 2021; Bostanghadiri et al. 2021). Results of BLAST and RDP analyses were confirmed by the Bayesian and ML phylogenetic analyses inferred on the basis of the obtained 16S rRNA gene sequences (Fig. 1). Even if 16S rRNA gene sequence analysis limited the ability to distinguish taxa at the species level (Dunlap 2019; Patel and Gupta 2020), the *Bacillus* core species (Subtilis and Cereus clade) and the new reclassified *Peribacillus* (Simplex clade) and *Neobacillus* (Niacini clade) (Patel and Gupta 2020) were clearly figured out. In detail, strains I13, I15–I17, O1, and O3 clustered within a well-supported clade (Bayesian posterior probabilities, BPP = 1 and aLRT = 1) together with *Bacillus subtilis subtilis* strain NCB 3610 (MK559753^T^), *Bacillus tequilensis* strain KCTC 13,622 (MW009674^T^) and *Bacillus velezensis* strain CR-502 (MK745998). Among this species, *Bacillus velezensis* and *Bacillus tequilensis* have an important role in agriculture due to their plant growth-promotion activity (Dunlap 2019; Kang et al. 2019; Oleńska et al. 2020), but their presence in commercial biostimulant or biofertilizer is underestimated due to misleading taxonomic attribution (Dunlap 2019).

**Table 2:** Results of BLAST and RDP analyses of partial 16S rRNA gene of bacterial isolated from commercial

Group	Isolate	ENA accession n	Family	RDP classifier assignment	GeneBank closest relative	% identity
	I 3	FR997860	*Enterobacteriaceae*	*Franconibacter*	*Cronobacter muytjensii* ATCC 51,329 (NR_118088)	95.52
	I 7	FR997864		*Franconibacter*	*Franconibacter helveticus* strain LMG 23,732 (NR_104980)	97.90
	O 2	FR997874		*Franconibacter*	*Enterobacter hormaechei* subsp. xiangfangensis strain LMG 27,195 (MZ540786^T^)	96.01
	I 14	FR997869		*Franconibacter*	*Enterobacter hormaechei* subsp. xiangfangensis strain LMG 27,195 (MZ540786^T^)	96.08
*Gammaproteobacteria*	I 6	FR997863	*Xanthomonadaceae*	*Stenotrophomonas*	*Stenotrophomonas maltophilia* strain MTCC 434 *(*MZ490578^T^)	98.61
	I 1	FR997858	*Bacillaceae*	*Bacillus*	*Bacillus paramycoides* strain NH24A2 (MT256266^T^)	97.35
	I13	FR997868		*Bacillus*	*Bacillus tequilensis* strain KCTC 13,622 (MW009674^T^)	99.33
					*Bacillus subtilis* subsp. subtilis strain NCIB 3610 (MK559753^T^)	99.10
	I 15	FR997870		*Bacillus*	*Bacillus tequilensis* strain KCTC 13,622 (MW009674^T^)	99.06
					*Bacillus velezensis* strain CR-502 (MK745998^T^)	99.06
	I 16	FR997871		*Bacillus*	*Bacillus tequilensis* strain KCTC 13,622 (MW009674^T^)	98.91
					*Bacillus velezensis* strain CR-502 (MK745998^T^)	98.91
	I 17	FR997872		*Bacillus*	*Bacillus tequilensis* strain KCTC 13,622 (MW009674^T^)	99.31
					*Bacillus subtilis* subsp. subtilis strain NCIB 3610 (MK559753^T^)	98.77
	O 1	FR997873		*Bacillus*	*Bacillus velezensis* strain CR-502 (MK745998^T^)	99.87
					*Bacillus tequilensis* strain KCTC 13,622 (MW009674^T^)	99.33
	O 3	FR997875		*Bacillus*	*Bacillus velezensis* strain CR-502 (MK745998^T^)	99.27
					*Bacillus tequilensis* strain KCTC 13,622 (MW009674^T^)	98.70
					*Bacillus subtilis* subsp. subtilis strain NCIB 3610 (MK559753^T^)	98.70
	I 2	FR997859		*Neobacillus*	*Bacillus drentensis* strain NBRC 102,427 (NR_114085)	97.50
					*Bacillus cucumis* strain AP-6 (NR_148626)	97.05
	I 4	FR997861		*Neobacillus*	*Bacillus niacini* strain NBRC 15,566 (NR_113777)	99.28
					*Bacillus drentensis* strain NBRC 102,427 (NR_114085)	98.68
					*Bacillus vireti* strain R-15447 (NR_025590)	98.32
	I 5	FR997862		*Peribacillus*	*[Brevibacterium] frigoritolerans* strain DSM 8801 (MK424281^T^)	99.78
	I 8	FR997865		*Peribacillus*	*[Brevibacterium] frigoritolerans* strain DSM 8801 (T) (MK424281^T^)	99.47
	I 9	FR997866		*Peribacillus*	*[Brevibacterium] frigoritolerans* strain DSM 8801 (T) (MK424281^T^)	99.31
*Firmicutes*	I10	FR997867		*Peribacillus*	*[Brevibacterium] frigoritolerans* strain DSM 8801 (T) (MK424281^T^)	99.51

^T^ Type strain

Strain I1 clustered within a well-supported (BPP = 1 and aLRT = 1) clade with *Bacillus acidiceler* (DQ374637), resulted sister of the previously reported *Bacillus* clade plus *Bacillus paramycoides* strain NH24A2 (MT256266^T^) and *Bacillus cereus* ATCC11778 (AF290546), the reference strain of Cereus clade. This clade includes human pathogens such as *B. anthracis*, but also *B. thuringiensis* a well-characterized biopesticide. Recently, the enteropathogenic potential of *Bacillus thuringiensis* isolates from biopesticides was assessed underlining the increasing risk for health consumers (Schwenk et al. 2020). Further studies will be carried out to ascertain the enteropathogenic potential of the strain I1 that grouped with bacterial species of Cereus clade.

The two isolated *Neobacillus* strains, viz., I2 and I4, clustered with *Neobacillus niacini* strain (MW243014) and of *Neobacillus drentensis* (MW243007), respectively. This genus was recently defined and included also *B. vireti* and *B. cucumis* (Patel and Gupta 2020) as reported in the here presented phylogenetic analyses (Fig. 1) *Peribacillus* strains (I5, I8–I10) grouped together with *Peribacillus simplex* (MG645295) and *Brevibacillus frigoritolerans* strain DSM 8801 (MK424281^T^), BPP support of 0.93 and aLRT of 0.79. *Peribacillus* and *Neobacillus* species were isolated from different environment such as soil and plant roots (Patel and Gupta 2020) but also from human gut and skin (Celandroni et al. 2016; Patel and Gupta 2020).

### Swimming motility

The effectiveness of microbial-based biostimulants is related to many factors such as their ability to colonize, survive, and proliferate for a considerable time inside and/or on plant tissues in the presence of indigenous microflora and, at the same time, directly or indirectly antagonize phytopathogens (Lugtenberg and Kamilova 2009; Pathania et al. 2020). The human and plant pathogens shared with beneficial bacteria the colonization process of the host including adhesion, invasion, and establishment. Bacterial movement strategies are the key point of this process (Vicario et al. 2015). Moreover, motility behavior of the bacterial isolates is considered as a crucial prerequisites for the onset of the diarrheal disease (Ghelardi et al. 2002; Mazzantini et al. 2016). The ability of the bacteria isolates to move as a single cell was evaluated at two different temperatures measuring the halo formed around the colony. Only three isolates molecularly identified as *Franconibacter* sp. isolate I3, isolate I7, isolate O2 and *Peribacillus* sp. isolate I10 did not show a swimming-related behavior*.* The non-swarming attitude in *Bacillus simplex* was also reported in other studies (Celandroni et al. 2016; Patel and Gupta 2020). The majority of the isolates, 14 out of 18, had the higher motility at 37 °C. In detail, *Bacillus* sp. (isolates I13, I15, I16, I17, O1, and O3), and *Franconibacter* sp. isolate I14 reached the plate border in 24 h at both temperatures. On the other hand, Bacillus sp. isolate I1 and *Franconibacter* sp. isolate O2 had higher motility, respectively, at 30 °C and 37 °C (Table 3). All the isolates showed a radial halo at 30 °C except *Bacillus* sp. isolate I1 that showed a dendritic halo. The dendritic morphology of the halo was also acquired by *Franconibacter* sp. isolate I14 and *Bacillus* sp. isolate I16, O1 and O3 at 37 °C (data not shown). Thus, it can be concluded that the swimming motility of the bacteria isolated from commercial biostimulants is partially temperature-dependent, but highly genus specific. Moreover, the dendritic motility was reported in human pathogens such as *Staphylococcus aureus* (Pollitt et al. 2015),* Pseudomonas aeruginosa* and *Bacillus subtilis* (Kearns 2010).

**Table 3:** Swimming motility of single strains analyzed on TrM plates (1% tryptone, 0.5% NaCl, 0.25% agar) after 24 h incubation at 30 °C and 37 C

Commercial product	Species	Isolates	Halo at 30 °C (Ø mm)	Halo at 37 °C (Ø mm)
Fertilizer with action on plant	*Bacillus* sp.	I1	15	1
	*Bacillus* sp.	I13	50	50
	*Bacillus* sp.	I15	50	50
	*Bacillus* sp.	I16	50	50
	*Bacillus* sp.	I17	50	50
	*Neobacillus* sp.	I2	1	9
	*Neobacillus* sp.	I4	1	7
	*Peribacillus* sp	I5	2	6
	*Peribacillus* sp.	I8	n.d	11
	*Peribacillus* sp.	I9	5	1
	*Peribacillus* sp.	I10	1	2
	*Stenotrophomonas* sp.	I6	1	7
	*Franconibacter* sp.	I3	1	1
	*Franconibacter* sp.	I14	50	50
	*Franconibacter* sp	I7	1	0
Fertilizer with action on soil	*Bacillus* sp.	O1	50	50
	*Bacillus* sp.	O3	50	50
	*Franconibacter* sp.	O2	4	50

The data represent growth halo 525 diameters (Ø mm)

### Swarming motility and biofilm formation

The collective cells movement on solid media was evaluated at 30 °C and 37 °C at different time points. All the tested isolates showed a swarming behavior after 48 h at 30 °C, while seven isolates started to swarm after 24 h at 37 °C (data not shown). On the other hand, *Neobacillus* sp. isolate I2, I4, and *Franconibacter* sp. strain I7 were characterized by lag period of 96 h of non-motile behavior. Only two isolates of *Peribacillus* sp. displayed a bull’s-eye phenotype at 37 °C.

**Fig. 2:**
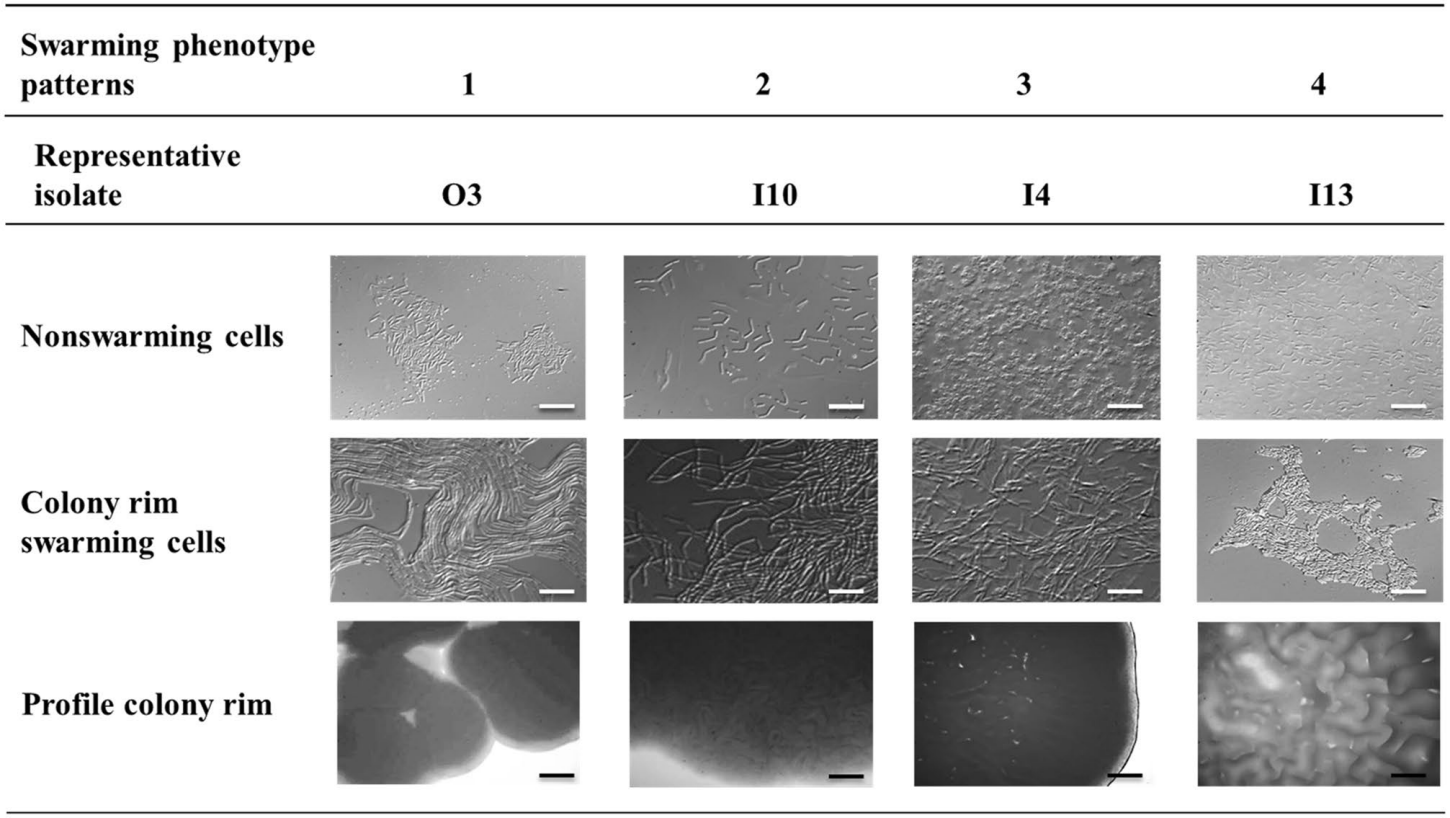
Morphological swarming cells’ traits and swarming phenotype patterns analyzed by light microscopy, 48 h after incubation. Strains grown on TrA plates (1% tryptone, 0.5% NaCl, 0.7% agar); white bars = 10 µm; black bars = 250 µm

**Fig. 3:**
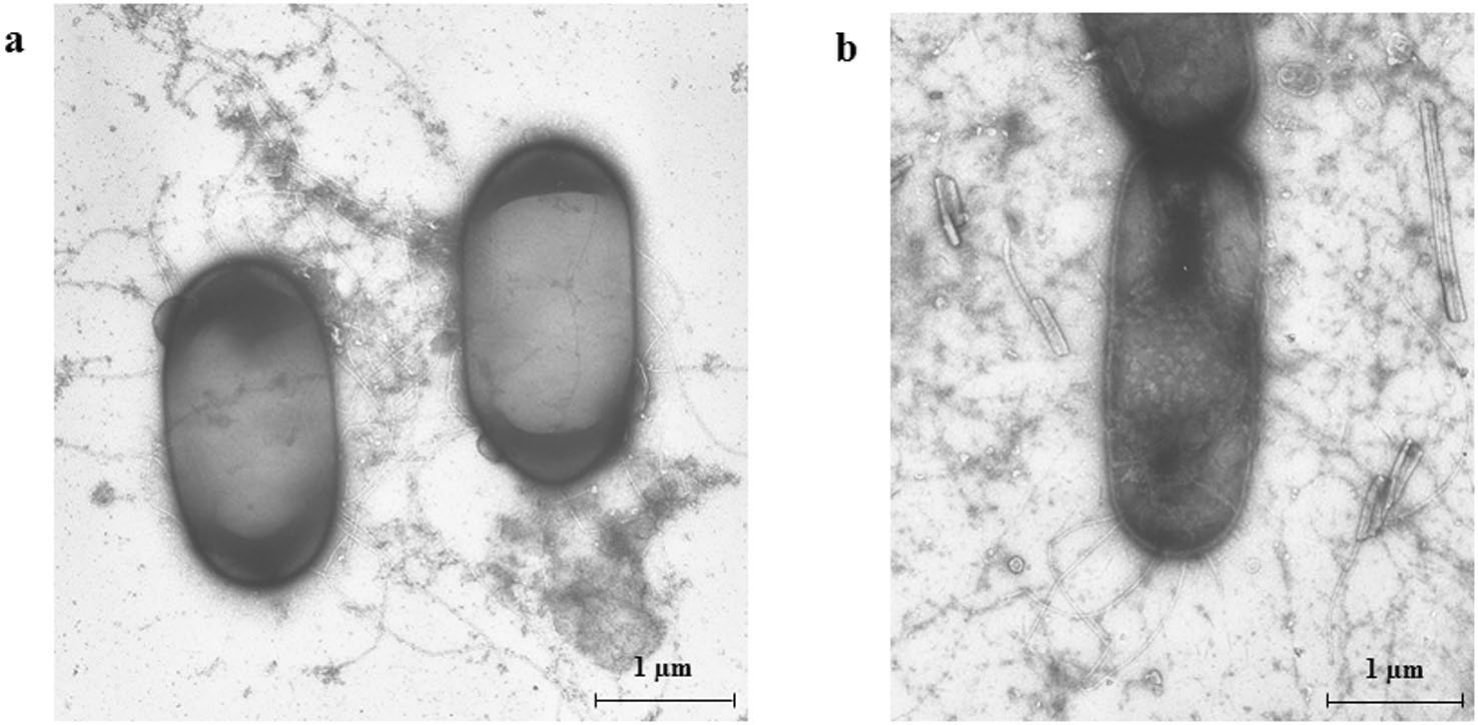
Morphological traits of isolate O3 observed by TEM after uranyl staining. **a** Short cells grown on TSA plates. **b** Elongated and chained swarm cells on TrA plates (0.7% agar). The cells were picked up from the colony rim after 48 h incubation at 30 °C

The isolates were clustered in four different morphotypes on the basis of colony rim (Fig. 2). In the first morphotype, the colony rim cells were long, filamentous swarm ones that maintained close cell-to-cell contact along their long axis while coordinately moving outward from the colony border in groups or rafts of tightly bound cells (Fig. 2). The rim cells of the second and third morphotype shared the elongated swarm cells and the cell migration all around the colony but differed in the cell density (Fig. 2). The fourth morphotype was associated to dendritic colony with a wavy rim composed by swarming cell in closed contact along their long axis (Fig. 2). Different bacteria changes their morphology substantially while transiting from a planktonic to a swarmer cell, as they become more elongated and their number of flagella significantly increase (Jones et al. 2004; Tuson et al. 2013). Electron microscopy showed an elongated morphology of *Bacillus* sp. isolate O3 swarmer cell (Fig. 3).

Flagellum-driven motility, such as swimming or swarming, may facilitate the colonization of different niches or the invasion of human host cellular barriers (Partridge and Harshey 2013). As previously reported for *B. cereus*, *B. pumilus*, and *B. licheniformis* (Celandroni et al. 2016), the high frequency of swimming- and swarming-proficient isolates in our collection suggests that these behaviors could contribute both to the capacity of these strains to colonize plant surfaces and/or to potentially establish an infection in humans.

**Fig. 4:**
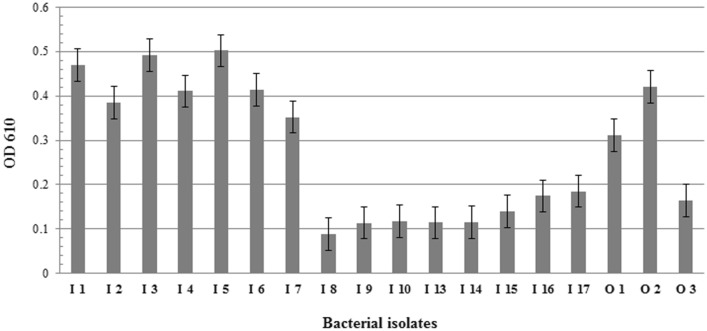
Biofilm formation quantified by the CV assay. The threshold for biofilm formation (solid line) is equal to the background signal plus two times the standard deviation (OD = 0.21). Values higher than the threshold level were considered positive for biofilm formation

The biofilm-formation assay was performed in LB medium. *Bacillus* sp. isolate I1, O1, all *Neobacillus* sp., *Peribacillus* sp. isolate I5 and *Franconibacter* sp. isolate I3, I7, and O2 produced biofilm in the tested condition (Fig. 4). On the other hand, in agreement with strong swarming behavior *Peribacillus* sp., *Bacillus* sp. (isolate I13, I15, I16, I17 and O3) and *Franconibacter* sp. isolate I14 were not able to form a biofilm community. The ability of the bacteria to form a biofilm is a common strategy to reduce the effects of biotic and abiotic stress (Costerton et al. 1995) but also a characteristic trait of PGPR and bacterial human pathogens.

### Pathogenic potential

The presence of virulence genes *sph*, as well as *nheA*, *nheB* and *nheC* genes encoding the sphingomyelinase and the three components of NHE, respectively, was evaluated by PCR amplification with available specific primers (Ghelardi et al. 2002). All these genes were detected in *Bacillus cereus* here used as control (Table 4). None of the isolates had the sphingomyelinase related gene while *nheA* was detected in two *Peribacillus* sp. isolates (I8 and I10), *Bacillus *sp*.* isolate I13, *Franconibacter* sp. isolate I3, I14, and *Stenotrophomonas maltophilia* isolate I6. Even if *Peribacillus* sp. isolates I8 and *Franconibacter* sp. isolate I14 had *nheA* and *nheC* genes, they had not the *nheB*-related genes as all the other isolates. Sequences of the NHE encoding genes were previously detected in *B. licheniformis*,* B. simplex*,* B. subtilis*, and *Paenibacillus* spp. (De Bellis et al. 2015). Interestingly, in this work, *B*. *cereus*-like toxin genes were found in the genus *Cronobacter* reported as food-borne pathogens capable of causing intestinal diseases (Jang et al. 2020). Since the full cytotoxic activity of non-hemolytic enterotoxin complex (NHE) required *nheA*, *nheB*, and *nheC* genes (Heilkenbrinker et al. 2013), *Peribacillus* sp. isolates I8 and *Franconibacter* sp. isolate I14 probably are not able to cause diarrheal type of food poisoning. As reported by Celandroni and colleagues (Celandroni et al. 2016), gene transfer or evolutionary mechanisms could explain the presence of such sequences in non-*B. cereus* species.

**Table 4:** Presence/absence of virulence and toxins/enzyme-related genes in bacteria isolated from two market biostimulants

Species	Isolate	Virulence genes				Toxins/enzyme			
		*sph*	*nheA*	*nheB*	*nheC*	*bceT*	*entS*	*entFM*	*piplc*
*Bacillus* sp.	I1	−	−	−	−	−	−	−	−
*Bacillus* sp.	I13	−	+	−	−	−	−	−	−
*Bacillus* sp.	I15	−	−	−	−	−	−	−	−
*Bacillus* sp.	I16	−	−	−	−	−	−	−	−
*Bacillus* sp.	I17	−	−	−	−	−	−	−	−
*Bacillus* sp.	O1	−	−	−	−	−	−	−	−
*Bacillus* sp.	O3	−	−	−	−	−	−	−	−
*Neobacillus* sp.	I2	−	−	−	−	−	−	−	−
*Neobacillus *sp*.*	I4	−	−	−	−	+	−	−	−
*Peribacillus* sp.	I5	−	−	−	−	+	−	−	−
*Peribacillus* sp.	I8	−	+	−	+	−	−	−	−
*Peribacillus* sp.	I9	−	−	−	−	−	−	−	−
*Peribacillus* sp.	I10	−	+	−	−	−	−	−	−
*Franconibacter* sp.	I7	−	−	−	−	−	−	−	−
*Franconibacter* sp.	I3	−	+	−	−	+	−	−	−
*Franconibacter* sp.	O2	−	−	−	−	−	−	−	−
*Franconibacter* sp.	I14	−	+	−	+	+	−	−	−
*Stenotrophomonas* sp.	I6	−	+	−	−	+	−	−	−
*Bacillus cereus* ATCC (control)		+	+	+	+	+	+	+	+

In addition, enterotoxin FM (EntFM), enterotoxin T (BceT), and enterotoxin S (EntS) were also analyzed out of the *B. cereus *sensu lato group. As reported in the literature, EntFM and BceT (Bhat et al. 2013; Mohkam et al. 2020; Heo et al. 2021) were not detected in the *Bacillus* isolates. Interestingly, *Bce*T was detected in *Peribacillus*, *Neobacillus*, and *Franconibacter* isolates, and in *Stenotrophomonas* sp.

## Conclusion

Functional tools for evaluating the pathogenic potential are urgently needed to predict hazardous potential of biostimulants.

In this study, cultivation-dependent methods combined to phylogenetic analysis showed the presence of a heterogeneous group of bacterial species in some biostimulants available on the market. Most of them belong to the genus *Bacillus* and newly defined *Neobacillus* and *Peribacillus*. Despite these bacteria are commonly considered soil-related organisms and PGPR, some of them (isolate I13 and O1) appear sufficiently equipped of virulence properties that could allow them to behave as pathogens/opportunistic pathogens for humans. Unexpectedly, also the genus *Franconibacter* and *Stenotrophomonas* were isolated from the biostimulants. These genera are reported in close association with plants (Ryan et al. 2009; Zhang et al. 2020), but *Stenotrophomonas*, particularly, shows a broad activity ranging from PGPR, bioremediation to multidrug-resistance human pathogens (Ryan et al. 2009; Bostanghadiri et al. 2021; Jiang et al. 2021).

In conclusion, the presented approach is useful to easily identify the bacteria present in undeclared rhizosphere bacteria-based formulations. Even more importantly, the combined analysis of motility and ability to form biofilms with the presence of toxin-encoding genes allows to discriminate between PGPR attitude and human pathogen potentiality. This discrimination could be the starting point for the development of a protocol to evaluate the hazardous potential of biostimulant products offering complete information about the safety of the applied strains and formulations to be implemented in routine biostimulant production to safeguard human and environmental health.

## Supplementary Information

Below is the link to the electronic supplementary material.

Supplementary file1 (XLSX 11 KB)

## Data Availability

16 rRNA gene sequences were deposited in the ‘European Nucleotide Archive’ and they will be permanently available from the ENA browser at http://www.ebi.ac.uk/ena/data/view/FR997858-FR997875
